# Surgical treatment for complete resection of transthoracic and abdominal schwannoma with diaphragm invasion using the transthoracic approach: a case report and literature review

**DOI:** 10.3389/fonc.2024.1467523

**Published:** 2024-11-29

**Authors:** Wen-jun Zhang, Li-song Pang, Dong-xia Hu, Yi-guan Le

**Affiliations:** ^1^ Department of Rehabilitation Medicine, The Second Affiliated Hospital, Jiangxi Medical College, Nanchang University, Nanchang, Jiangxi, China; ^2^ Gastrointestinal Surgery, The Second Affiliated Hospital, Jiangxi Medical College, Nanchang University, Nanchang, Jiangxi, China

**Keywords:** mode of operation, schwannoma, diaphragm injury, surgery, treatment

## Abstract

**Background:**

The removal of schwannomas involving the chest and abdominal cavities is difficult, which requires a high level of overall proficiency and technical expertise from surgeons. Therefore, this study explored a safe and feasible surgical method for the complete resection of this type of tumor.

**Methods:**

We collected the medical records of a 44-year-old female patient with space-occupying lesions near the thoracic vertebrae.

**Results:**

The transthoracic approach could effectively expose the relationship between the tumor and adjacent tissues, reduce the patient’s additional injury, completely remove the tumor tissue, and repair the diaphragm defect well. The amount of blood loss during the operation was approximately 600 ml. After the operation, there was a small amount of pleural effusion, the patient recovered well, and no other complications occurred. Through follow-up for 3 months after the surgery, the patient had no other complications and his physical condition had recovered.

**Conclusions:**

For patients with transthoracic and abdominal schwannoma complicated by diaphragmatic invasion, performing tumor resection using the transabdominal approach may be difficult, but the transthoracic approach may be a safe and feasible surgical option.

## Introduction

Schwannomas, which develop from Schwann cells of the nerve sheath, are mesenchymal tumors, mainly located in the peripheral nerve or central nervous system (1–3). They are the most common extramedullary intradural tumors in the thoracic spine. This kind of tumor is often accompanied by degenerative changes such as bleeding, calcification, and fibrosis (4, 5). Although it is a histologically benign tumor, we should not underestimate the damage caused by compression and destruction of adjacent structures, as well as possible malignant transformation (1, 6, 7). Schwannoma is not uncommon in the spine, but it is clinically rare in patients with schwannoma invading the diaphragm and protruding into the abdominal cavity.

Complete resection of schwannoma needs extensive experience and surgical techniques, requiring the operator to have a clear and accurate grasp of the anatomical structure of thoracic and abdominal tissues (8, 9). Especially when the tumor invades the diaphragm and abdominal organs across the chest and abdominal cavity, it is a challenge for the operator to choose the best approach to expose the relationship between the tumor and the surrounding tissue and to improve the intraoperative visual field (10). The choice of an inappropriate approach may lead to a smaller operating space during the operation, and it may be difficult to expose the relationship between the tumor and the surrounding tissue structure, which may lead to the destruction of the integrity of the tumor tissue, the increase of the probability of tissue injury, and the increase of bleeding volume during the operation (11, 12). Therefore, it is very important to make an overall evaluation of the patient’s condition before the operation, choose a safe and feasible method, fully expose the tumor, increase the intraoperative visual field, and reduce the incidence of intraoperative and postoperative complications and accidents. All these bring great challenges to surgeons.

In this study, we reported a very challenging case. The tumor spanned the abdominal cavity and thoracic cavity and invaded the diaphragm. It was difficult to locate the tumor body on imaging, which led to some errors in our preoperative judgment. A complete evaluation was performed again during the operation and the tumor was finally removed. By sharing this experience, we hope to provide valuable help to other surgeons in the treatment of similar diseases, avoid detours, and adopt safe and feasible resection methods through the transthoracic approach.

## General condition of the patient

**Figure 1:**
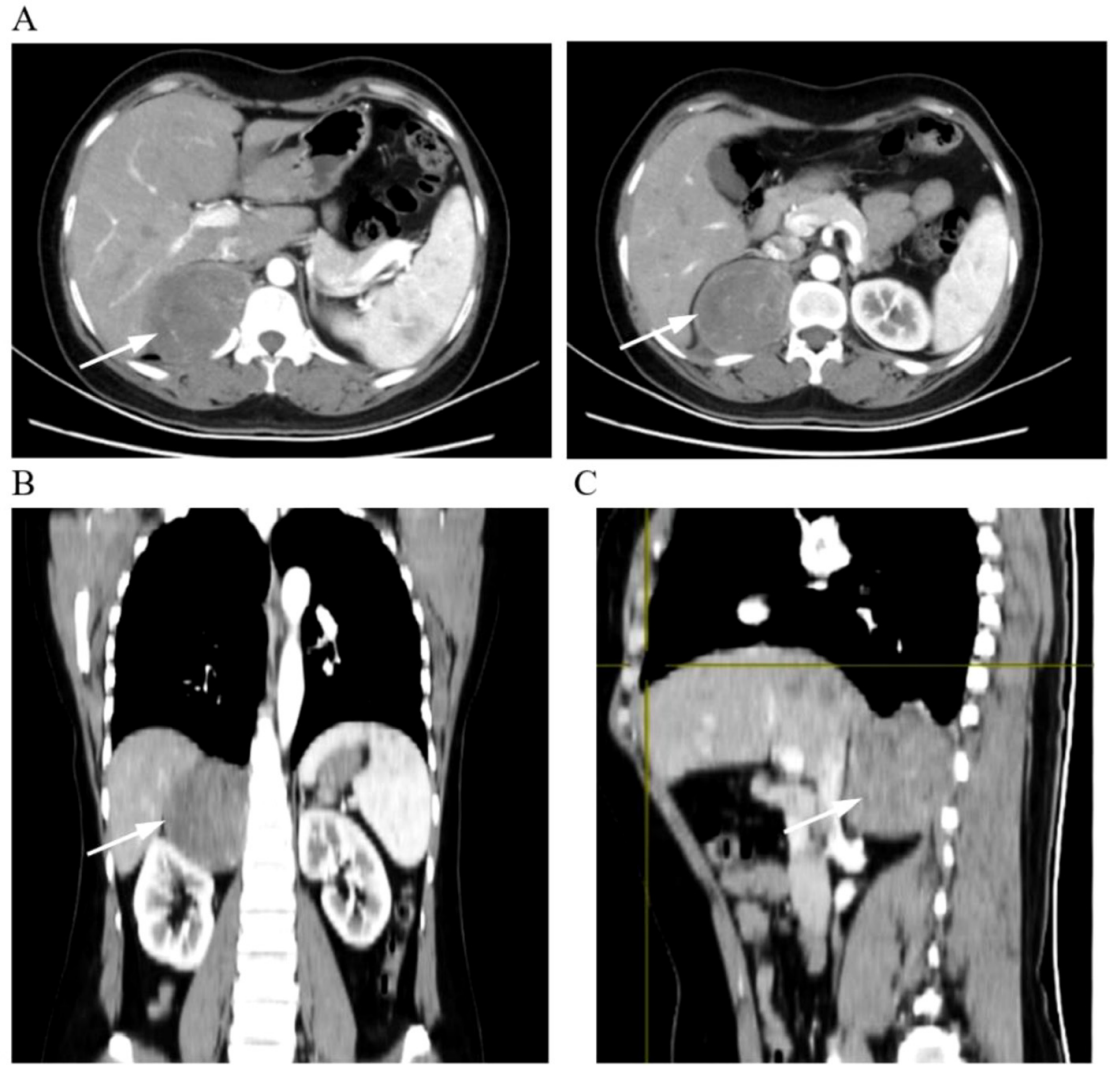
Representative images of CT scans. **(A)** Cross-sectional enhanced scan. **(B)** Coronal scan. **(C)** Sagittal scan. The arrows in the picture show the tumor tissue.

**Figure 2:**
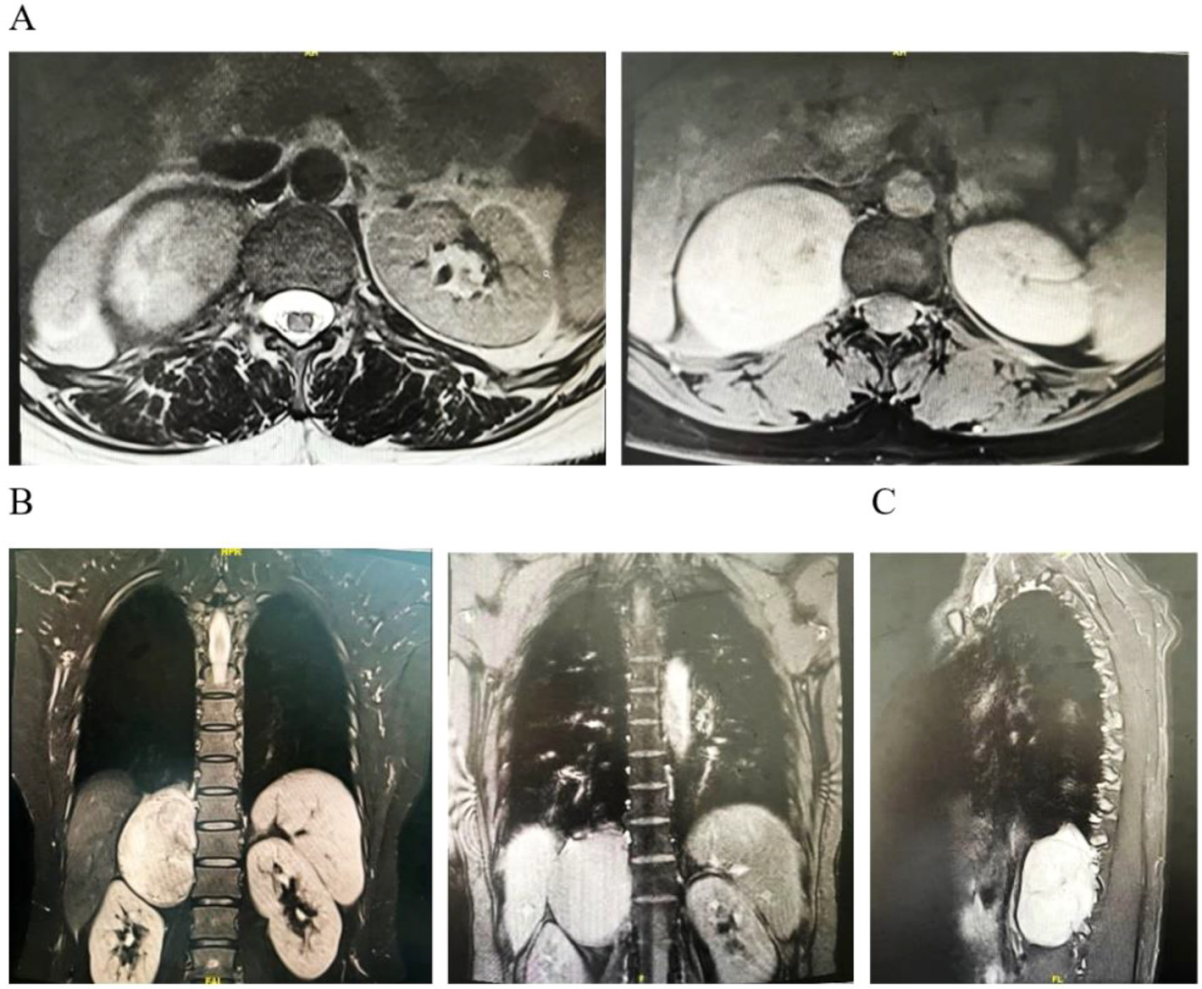
Representative images of MRI scans. **(A)** Cross-sectional scan. **(B)** Coronal scan. **(C)** Sagittal scan.

A 44-year-old woman had no symptoms of nausea and vomiting, dizziness and headache, chest tightness, shortness of breath, and abdominal pain as shown by the color Doppler ultrasound findings. CT examination showed a fusiform soft tissue density shadow around the vertebra on the right side of T11-L2, the size of which was approximately 70 × 60 × 79 mm. A further enhanced scan showed mild to moderate uneven enhancement of soft tissue and multiple small vessels (**Figure 1**). In order to better display and judge the tumor condition, the patient was further examined by MRI. The results showed a fusiform soft tissue mass beside the vertebra on the right side of T11-L2, slightly longer T1 and T2 signals, an uneven internal signal, and flake-long T1 and T2 signals. The tumor’s maximum cross-section measured 70 × 55 × 86 mm, with local growth toward the right intervertebral foramen, and there were compression and displacement of adjacent abdominal organs. Enhancement showed that the mass had obvious inhomogeneous enhancement, and there was a flaky non-enhanced area inside (**Figure 2**). The results of the physical examination were as follows: temperature, 36.1°C; respiratory rate, 20 breaths per minute; blood pressure, 148/86 mmHg; and pulse, 70 beats per minute. The patient’s tests included blood routine, liver function, renal function, electrolytes, blood coagulation, ECG, and lung function. These functions were basically normal.

## Tumor abnormalities found by the transabdominal approach

Routine blood work was performed before the operation, and the patient was transferred to the operating room. Doctors, nurses, and anesthesiologists checked the patient’s information. The patient was placed on the operating table in the left lying position and received endotracheal intubation for general anesthesia. After successful anesthesia, routine disinfection and sheet laying were performed. A 1.5-cm incision was made at the anterior edge of the inferior psoas major muscle at the 12th costal edge of the posterior axillary line of the patient. We used vascular forceps to separate the subcutaneous tissue, muscle layer, and lumbar dorsal fascia and placed the self-made airbag into the retroperitoneal cavity. To enlarge the posterior peritoneal cavity, 500 ml was injected and retained for 5 min. Then, trocars measuring 12 mm and 10 mm were positioned at the subcostal margin of the anterior axillary line and at two transverse fingers above the iliac ridge of the midaxillary line, respectively. Moreover, a 5-mm trocar was inserted into the posterior axillary line. Then, the pneumoperitoneum machine was connected, the pressure was maintained at 12–15 mmHg, the mirror sheath was inserted at the iliac incision, and the other two incisions were inserted into the operation piece. Gerota’s fascia was cut open with a separation rod and an ultrasonic knife, and the ventral and dorsal sides of the superior margin of the kidney were dissociated between the fascia and the perirenal fat space, exposing the right adrenal area. It was found that there was a space occupying between the right adrenal gland and the lumbago dorsal muscle, indicating that it was smooth and had a size of approximately 6 × 7 cm, and that the tumor was clearly defined from the right adrenal gland and kidney.

We opened the external capsule of the tumor and found that the main body of the tumor was located in the thoracic cavity. We found that it was difficult to completely expose the tumor and remove it. Continuing the operation may lead to the rupture of the tumor and other additional injuries. Therefore, in order to better expose the tumor and remove it completely, we asked the director of thoracic surgery for consultation and changed the approach to remove the tumor through a combined transthoracic approach.

## Complete resection of the tumor and repair of the diaphragm using the transthoracic approach

After the double-lumen bronchial intubation was successful, the chest was routinely disinfected and toweled. A 3-cm incision was made at the sixth intercostal space on the right side, allowing direct entry of the thoracoscope into the thoracic cavity, and the tumor was revealed to be located at the right diaphragmatic angle. As the visual field exposure was still found to be poor, a perforation was taken at the eighth intercostal space of the right axillary midline, and it was found that the tumor was closely related to the diaphragm and part of it protruded to the abdominal cavity. At this time, the operation field of vision had been well exposed. The tumor and its surrounding tissue along the tumor capsule were slowly dissociated with an ultrasonic knife, fully exposing the tumor, and the tumor was found to be rich in blood vessels. Intraoperative bleeding was stopped using an ultrasonic scalpel and an electric hook. After complete exposure and resection of the tumor, it was found that there was a long fissure of 5 cm in the diaphragm, and then a non-absorbable thread was used to suture the diaphragm intermittently. Finally, the bleeding was stopped thoroughly, the chest and abdominal cavity were washed, a drainage tube was placed at the puncture hole of the chest cavity, bleeding due to the incision was stopped, and the chest cavity was closed.

The whole operation process was smooth, with satisfactory intraoperative anesthesia results and no additional injury. Because the tumor was rich in blood vessels, there might be blood osmosis during the isolation of the tumor, with an estimated blood loss of 600 ml. To address this, the patient received an infusion of 3 units of leukocyte-depleted suspended red blood cells and 200 ml of fresh frozen plasma. The specimen was examined by the family members and sent for examination. The patient was transferred to the recovery room under medical supervision.

The final pathological and macroscopic examination revealed that the tumor section was gray-white or grayish-yellow, solid, and tough, with local dark red bleeding, a clear tumor boundary, and a visible cystic wall-like tissue. Under a microscope, the tumor tissue was spindle-shaped, arranged in bundles, with mild cells and clear tumor boundaries. Schwannoma was the final diagnosis.

## Postoperation and discharge

**Figure 3:**
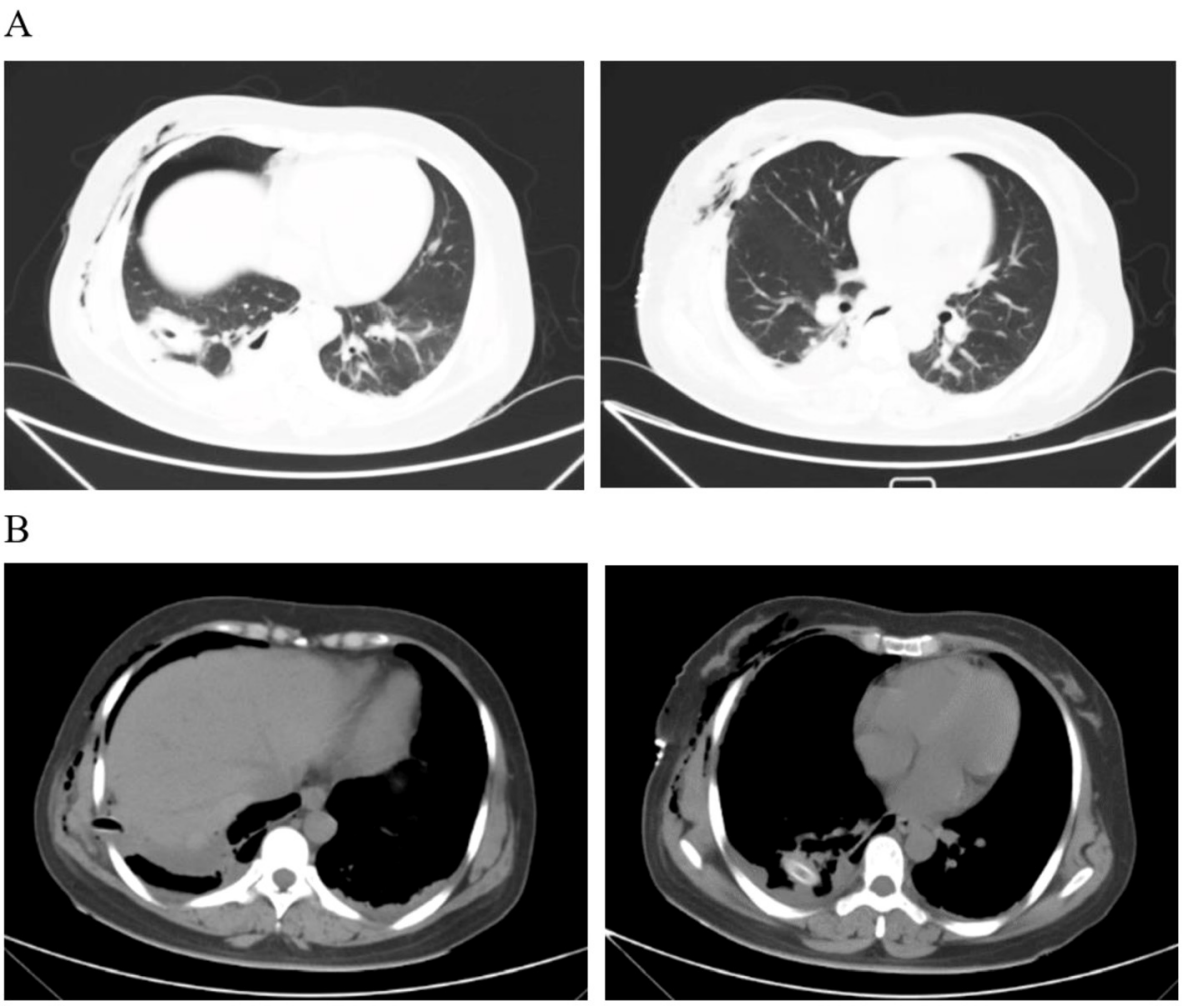
Pleural effusion was scanned by CT on the third day after the operation. **(A)** The lung window shows a little consolidation of the lungs. **(B)** The drainage tube can be seen in the mediastinal window, and a small amount of effusion can also be seen.

After the operation, the patient was given comprehensive treatment such as fasting, fluid replacement, nutritional support, stomach protection, aerosol inhalation, anti-infection, anticoagulation, and analgesia. Regular reexamination of blood routine, liver and kidney function, and electrolytes was conducted. The quantity, color, and character of the thoracic drainage tube were also monitored. CT examinations were performed on the third day after the operation. The results showed that the space-occupying lesions had been resected, and a small amount of effusion and pulmonary consolidation were seen in the right pleural cavity. Moreover, there was also a small amount of exudation in the abdominal cavity and perirenal cavity (**Figure 3**). There were no complications such as anastomotic leakage, chest tightness and shortness of breath, bleeding, abdominal distension, and abdominal pain. The patient recovered well and was discharged smoothly on the 12th day. Moreover, the patient was followed up for 3 months after discharge. The patient had no other related complications such as chest tightness, shortness of breath, fever, chills, abdominal distension, and abdominal pain, and his overall physical condition showed steady improvement.

## Discussion

Schwannomas are well-encapsulated, slow-growing tumors that originate from benign Schwann cells in the peripheral nerve sheath. Because of their rare and complex anatomical position, they are usually misdiagnosed during clinical evaluation (9, 13). Schwannomas can occur in many parts of the body, such as retroperitoneal (14), saphenous nerve (15), subarachnoid space (16), bone (17), posterior mediastinum (18), head and neck, and flexor muscles of extremities (19). They usually do not transfer but can cause serious problems in the nerves, blood vessels, and adjacent organs and tissues (20, 21). Sengul et al. reported a 63-year-old male patient who had an axillary painless mass for 4 months and was confirmed to be axillary schwannoma by histopathological examination and immunohistochemistry (13). Ali et al. reported a case of intraosseous neurilemmoma in the distal shaft of the fibula. The patient was an 18-year-old woman with pain in her right leg for 2 years. MRI showed oncolytic lesions which were differentiated from aneurysmal bone cysts. After resection of the tumor, it was diagnosed as intraosseous schwannoma by histopathological examination (22). All these suggest that schwannomas can occur in different parts of the body.

The posterior mediastinum is a potential space along the paraspinal groove or between the back of the pericardium and the vertebrae. This area is usually the most common location of neurogenic tumors. Tumors located in the posterior mediastinum rarely grow very large. The definite diagnosis and treatment of these tumors are the key to surgical resection. Schwannomas are neurogenic tumors that usually originate from the peripheral nerve and present as an asymptomatic solitary mass on imaging (17, 23). Depending on the structure or location of their origin, the diagnostic procedures and subsequent surgical plans and methods may change. The operation of schwannomas should be comprehensively evaluated. Detailed operation planning, adjuvant treatment, proper preoperative management, and follow-up should take into account the final histopathological features (18, 23, 24). However, in this study, the patient had a type of schwannoma that is very rare, making resection more difficult and requiring a rich clinical experience and a good anatomical basis. Preoperative evaluation and intraoperative differences led us to change the surgical plan. During the operation, we did not forcibly resect the tumor and separate the surrounding tissue structures, causing the tumor capsule to rupture and spread. Instead, we conducted a comprehensive evaluation again, changed and formulated the surgical plan, and performed a complete resection through the transthoracic approach. Therefore, it is very important to choose the appropriate surgical approach and scope, improve the operating space, better expose the tumor tissue, and improve the tumor resection rate. Through this experience, we found that it was difficult to expose the tumor tissue and remove the tumor that straddled the chest and abdominal cavity and invaded the diaphragm using the transabdominal approach. The choice and scope of the surgical approach had great advantages for the complete resection of the tumor and could reduce the time of operation and possible injury during the operation, which are very important for both operators and patients. In addition, for the preoperative evaluation and consideration of the surgical plans, it is very important to strengthen the preoperative planning and overall preoperative evaluation by using advanced imaging technologies such as 3D reconstruction to better assess the location of the tumor, show the adjacent relationship between the tumor and surrounding tissues, and plan the surgical approach. For the resection of this type of tumor, the surgeon’s comprehensive preoperative evaluation, intraoperative evaluation, intraoperative practice, choice of surgical methods, and experience are essential.

Furthermore, drawing from our years of clinical experience, we also have gained insights and techniques during surgery. We think that we should pay attention to several points during the operation. First, through imaging examination, we should fully and carefully discuss the relationship between the tumor and the surrounding tissues and organs and judge the source of the tumor and its main site. It is suggested that three-dimensional reconstruction should be used, so the whole picture of the tumor can be better displayed. Second, if the tumor is rich in blood vessels, this should not be a cause for alarm as this can be managed successfully through preoperative and intraoperative judgment by carefully separating the tumor tissue. If there is more vascular injury or bleeding, gauze can be used to stop the bleeding. If significant bleeding occurs, transfusion and blood products can be administered simultaneously through an open channel, and changes in vital signs such as urine volume and blood pressure can be closely monitored. Third, when removing tumor tissue, it is necessary to ensure the integrity of its capsule, avoid the residual tumor tissue, adopt sudden separation as far as possible, and carefully separate and expose it. It is worth mentioning that as schwannomas can compress and displace adjacent tissues, with the possibility of having tissue structure variations, careful attention is needed to distinguish them during the course of the operation to avoid unnecessary damage. Lastly, if there is limited experience in diaphragm repair and closure, it is recommended to ask a chest doctor to assist in suturing the diaphragm, preventing diaphragm rupture, diaphragmatic hernia, and negative impacts on the diaphragm and respiratory function.

## Data Availability

The original contributions presented in the study are included in the article/supplementary material. Further inquiries can be directed to the corresponding author.
